# Effect of presentation rate on auditory processing in Rett syndrome: event-related potential study

**DOI:** 10.1186/s13229-023-00566-1

**Published:** 2023-10-26

**Authors:** Daria Kostanian, Anna Rebreikina, Victoria Voinova, Olga Sysoeva

**Affiliations:** 1 Center for Cognitive Sciences, Sirius University of Science and Technology, Olympic Ave 1, Sochi, Russia 354340; 2 Laboratory of Human Higher Nervous Activity, Institute of Higher Nervous Activity and Neurophysiology of RAS, Moscow, Russia 117485; 3 Veltischev Research and Clinical Institute for Pediatrics of the Pirogov, Russian National Research Medical University, Ministry of Health of Russian Federation, Moscow, Russia 125412

**Keywords:** Rett syndrome, Auditory event-related potential (ERP), Presentation rate, Stimulus-specific adaptation

## Abstract

**Background:**

Rett syndrome (RS) is a rare neurodevelopmental disorder characterized by mutations in the *MECP2* gene. Patients with RS have severe motor abnormalities and are often unable to walk, use hands and speak. The preservation of perceptual and cognitive functions is hard to assess, while clinicians and care-givers point out that these patients need more time to process information than typically developing peers. Neurophysiological correlates of auditory processing have been also found to be distorted in RS, but sound presentation rates were relatively quick in these studies (stimulus onset asynchrony, SOA < 1000 ms). As auditory event-related potential (ERP) is typically increased with prolongation of SOA we aim to study if SOA prolongation might compensate for observed abnormalities.

**Methods:**

We presented a repetitive stimulus (1000 Hz) at three different SOAs of 900 ms, 1800 ms, and 3600 ms in children with RS (*N* = 24, Mean age = 9.0 ± 3.1) and their typical development (TD) peers (*N* = 27, Mean age = 9.7 ± 3.4) while recording 28-channels electroencephalogram, EEG. Some RS participants (*n* = 10) did not show clear ERP and were excluded from the analysis.

**Results:**

Major ERP components (here assessed as N1P1 and P2N1 peak-to-peak values) were smaller at SOA 900 than at longer SOAs in both groups, pointing out that the basic mechanism of adaptation in the auditory system is preserved in at least in RS patients with evident ERPs. At the same time the latencies of these components were significantly delayed in the RS than in TD. Moreover, late components (P2N1 and N2P2) were drastically reduced in Rett syndrome irrespective of the SOA, suggesting a largely affected mechanism of integration of upcoming sensory input with memory. Moreover, developmental stagnation of auditory ERP characterized patients with RS: absence of typical P2N1 enlargement and P1 and N1 shortening with age at least for shortest SOA.

**Limitations:**

We could not figure out the cause for the high percentage of no-evident ERP RS participants and our final sample of the RS group was rather small. Also, our study did not include a control clinical group.

**Conclusions:**

Thus, auditory ERPs inform us about abnormalities within auditory processing that cannot be fully overcomed by slowing presentation rate.

**Supplementary Information:**

The online version contains supplementary material available at 10.1186/s13229-023-00566-1.

## Background

Rett syndrome (RS) is a neurodevelopmental disorder associated with mutations in the X-linked gene *MECP2* [1]. This disease is characterized by a variety of physiological, motor, and cognitive deficits that usually follow relatively typical initial development [2]. After regression that occurs at about 6–18 months of age, most children with RS is non-verbal [3–5] and have severe problems with goal-oriented motor actions [6], making it hardly possible to use standard tools to assess their cognitive abilities. Thus, limited data exist on the specification of cognitive function including the ability to perceive and understand speech in RS.

Auditory event-related potentials (ERP) is a convenient tool for assessing the processing of auditory information in the brain, as it does not require participants attention and can be used in challenging populations. It consists of positive and negative components (P1, N1, P2, and N2) whose prominence depends on many parameters including rate of presentation [7–10]. In particular, the N1 and P2 components become larger as the inter-stimulus interval increases at least up to 12 s [11, 12]. Neuronal networks activated by the sounds do not come to their initial state immediately after sound termination but their activation fades slowly. If during this fading period the similar sound is presented it could not elicit the “full” response as the first stimuli have elicited but only its fraction (that might be also called adaptation). The more neurons return to their initial state the larger the response. That what we believe is captured by ERP modulation by the rate of presentation. These neurophysiological changes were linked to the decay of the stimulus’ memory representation as it corresponds with behavioral results in psychophysical experiments [11–13]. In general, faster presentation rate is better for integration information over many temporally segregated stimuli, while slower rate allows to process each stimuli thoroughly. However, the optimal limit for each type of analysis is varied across individuals. For example, adult dyslexics have faster memory trace decay than good readers that provide difficulties in integrating the information within several seconds while in autism the memory decay is longer putting less weight into the most recent event and increasing the ability to link together unrelated things [14].

In the current study we aim to examine modulation of auditory ERPs by the rate of stimulation in RS to dig into the mechanisms underlying Rett symptomatology. Previous studies have shown that auditory ERPs are altered in RS: its late components P2 and N2 almost absent when stimulus is presented at about one per second—typical rate of presentation in neurophysiological experiments [15, 16]. No study has examined the auditory ERPs in RS in response to more slowly presented stimuli with stimulus onset asynchrony longer than 2 s. However, as the speed of signal processing is reported to be low in RS as indicated with delayed ERP components [17–19] these patients might benefit from the slower presentation rate.

Assessing ERP modulation by presentation rates in RS provides information on the basic adaptation mechanism in this group that might be differentially preserved depending on the stage of auditory processing. In particular, P1 is linked to early processing of sensory information [20] and consistent with previous findings we do not expect any deficits in it with different presentation rates in RS. The N1 component, related to both sensory and cognitive processing, on the contrary, has been demonstrated to be especially sensitive to the SOA duration in neurotypical individuals [10]. This component has been preserved in RS for short SOAs [15], thus we believe it might also show typical enlargement with SOA prolongation. At the same time, P2 component, associated with consolidation of auditory memory [21], has been drastically reduced in RS with short SOA, and may recover to the typical level with slowing presentation rate, following the pattern seen in typically developing children. Meanwhile, N2, which has been associated with categorization [22–25], and suppression of irrelevant information [26], has also been reduced in RS with short SOAs [15]. While there is no data on N2 modulation by stimulus presentation rate in neurotypical samples, nobody examined this aspect in RS keeping the question of its potential recovery at slow presentation rate open.

Thus, we hypothesize that by increasing the stimulus onset asynchrony from 0.9 to 3.6 s we might see recovery of auditory ERP components in RS that will get more typical. Simultaneously we are assessing if the ERP components are modulated by the rate of tone presentation, getting insight into the mechanism of basic memory function in this group.

As both N1 and P2 components are modulated by the duration of the interstimulus interval in neurotypical individuals, there is a discussion concerning whether common or independent processes are behind this modulation [27]. From one side, a strong correlation between the amplitudes of N1 and P2 was shown in Pereira work [9], which might be indicative of the common mechanism underlying their modulations. However, fitting an adaptation model for each of these components demonstrates distinct dynamics of their adaptation [11]. As N1 and P2 components are differentially affected in the Rett syndrome ERPs, assessing the relationship between their amplitudes and their modulation also appears to be important.

## Methods

### Participants

*Rett syndrome group:* 24 children aged from 3 to 17 (mean age = 9.0 ± 3.1) with Rett syndrome participated in this study. They were recruited during clinical visits to the Research Clinical Institute of Pediatrics in Moscow, Russian Federation. The diagnosis was based on current diagnostic criteria and was confirmed clinically by a medical doctor specializing in this population (V.V.) as well as via genetic testing. All participants had a pathogenic variant in the *MECP2* gene. All participants were in the post-regression phase. Severity of RS was measured using the Rett syndrome severity scale (RSSS) [28]. This scale assesses individual parameters: frequency and severity of seizures, breathing irregularities, scoliosis, ability to walk, use of hands, speech and sleep.

More detailed characteristics of the sample, including the type of mutations, can be found in the Additional file 1: Table 1. All participants from this group were females as this rare disease affects mostly females.

*Typical development group:* Twenty-seven children aged from 2.5 to 16 (mean age = 9.7 ± 3.4) years without neurological, psychiatric disorders, mental and speech delays, or hearing problems according to parental reports. 8 out of TD participants were males.

Parents or legal representatives have given written consent to the children’s participation in the study, after the procedure was explained to them. Where capable children have given verbal consent to participate. The research procedure met the standards for research from the Helsinki Declaration of 1975 (Protocol 1 from 01.15.2020) and was approved by the ethical committees of IHNA and Nph RAS (Protocol №2 at April 30th, 2020) and Sirius University of Science and Technology amendment from April 15th, 2021.

The sample size was calculated using the G*power 3.1.3 program (Heinrich-Heine-University, Düsseldorf, Germany) with a statistical power of 80%, and a significance level of 0.05. To assess the correlation with symptom severity (anticipated effect size = 0.62, according to Sysoeva work [15]), the required sample size was 18 participants. To assess interaction effects between two groups with three levels of within-group factor (anticipated effect size = 0.3, according to approximate medium effect size), minimum total sample size should be 20 people.

### Experimental design

#### Stimuli

Pure tone 1000 Hz (duration: 100 ms, loudness: 65 dB) was presented in three experimental blocks with three different stimulus onset asynchrony (SOAs): 900, 1800, and 3600 ms. Stimuli with each type of SOA were presented in separate blocks. Each tone was presented 150 times for 1800-ms and 3600-ms SOA conditions, and 300 times for 900-ms SOA conditions. The large number of trials for the 900-ms SOA condition was a precaution to get sufficient number of epochs for averaging when epochs with motion and other artifacts are excluded and to be able to run other types of analysis. For the current analysis only the first 150 artifact-free epochs from 900-ms SOA condition were used to equate with other SOA conditions. We used 1000 Hz tone as previous studies [15, 29] pointed out that the deficits in ERP are present even at the level of pure tones analysis.

#### Procedure

Participants sat in a comfortable chair in a sound-attenuated room. Participants listened to sounds binaurally through earphones and watched a muted video of their or parent’s choice. They were instructed to ignore sounds and avoid moving. In case children were not able to follow instructions, the parent sat nearby and held the child if necessary to limit motor activity. In some cases, the child sat on the parent's lap. In the short breaks between blocks, participants can change their positions.

### EEG recordings

Electroencephalographic data were recorded using the NeuroTravel system with 28-scalp electrodes arranged according to the international 10–20 system guidelines ('Fp1', 'Fp2', 'F3', 'Fz', 'F4', 'F7', 'F8', 'Fc3', 'Fcz', 'Fc4', 'C3', 'Cz', 'C4', 'Cp3', 'Cpz', 'Cp4', 'P3', 'Pz', 'P4', 'Tp7', 'Tp8', 'T3', 'T4', 'T5', 'T6', 'O1', 'Oz', 'O2'). Linked earlobe electrodes were used as reference, and AFz as ground, and 0.01–70 Hz online filters were applied. The data were sampled at 500 Hz. The electrode impedances were below 10 kΩ.

### Data processing

EEG was filtered with 2–20 Hz offline filters. 2 Hz high-pass filter was used due to high amplitude delta oscillations evident in many participants with Rett syndrome. Application of this filter allows accepting more trials for signal averaging and consequently better signal-to-noise ratios. Using such high-pass filters can be associated with risks of artifactual effects [30] but mainly for the later components (N400 and P600) and absolute amplitude values. Considering peak-to-peak amplitude can help to avoid negative impact of filtration on the results. Despite the fact that the use of strong high-pass filters may lead to misleading results [31], using high-pass filters is common practice when considering ERP of participants with Rett syndrome (e.g. 3 Hz high-pass filters were used in Saby’s work [16]).

Bad channel interpolation was applied when necessary (0–2 channels per participant). Automatic raw data inspection with ± 400 μV thresholds was used for rejecting EEG segments with large artifacts, then for artifact (eye movements, muscle activity) rejection, the independent component analysis (ICA) was performed, in particular the ALICE platform was used [32]. The data were segmented into epochs starting 200 ms before a stimuli onset and lasting 500 ms after the onset. Automatic rejection of the bed segments with signals more than ± 100 μV was applied. ERPs were baseline corrected to -200 ms prestimulus intervals. Mean number of epochs to average for each participant was 150 (first 150 trials taken from 236 + 23 and 226 + 44 remaining after artifact rejections trials) for 900-ms SOA condition, 127 ± 14 and 123 ± 20 for 1800-ms, 123 + 18 and 118 + 18 for 3600-ms SOA conditions for TD and RS, respectively.

**Fig. 1:**
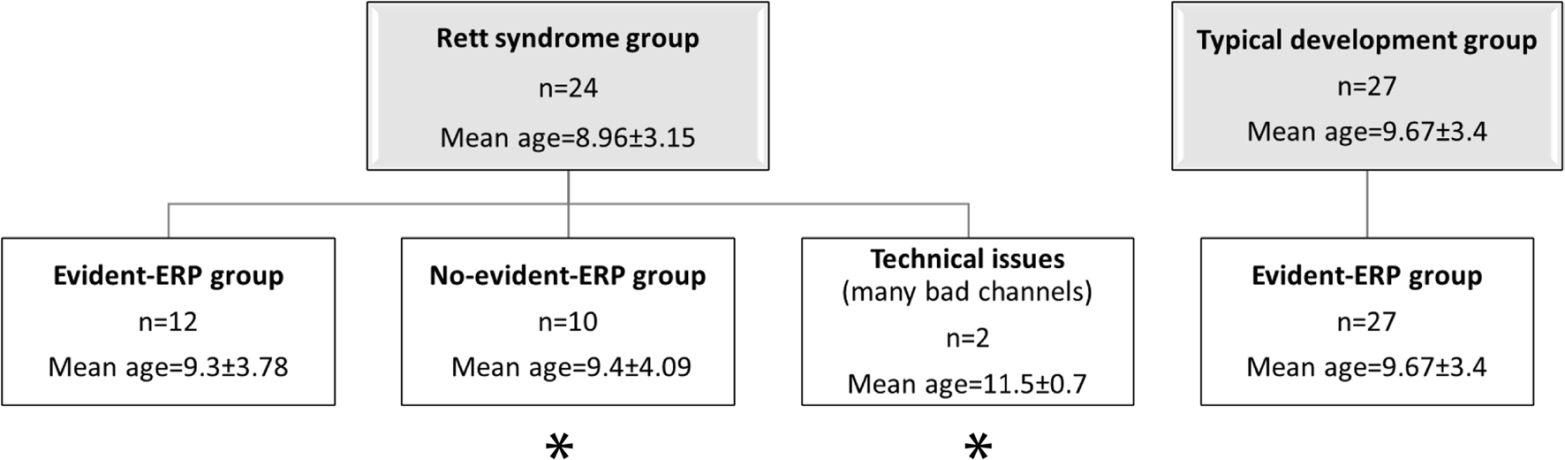
Participants’ grouping that includes information about detectability of Event-related potential (ERP) (groups with * were excluded from the analysis)

The FCz channel was chosen for the analysis, according to the literature as the auditory cortex response is observed in this area [7]. Also, for this channel ERP components were more pronounced and less affected by artifacts. Some participants from the RS group did not have a clear peaks’ morphology, so it was hard to estimate absolute peak amplitude and latency. Thus all participants were evaluated on the prominence of ERP’s components by two independent experts that were blind to the diagnosys (Fig. 1). All participants from TD group had a clearly identified ERP component, while participants from the RS group were divided into two groups: evident-ERP (*N* = 12) and no-evident-ERP components groups (*N* = 10). Two RS participants' data were excluded from the analysis because of a high number of bad channels. In no-evident ERP RS groups average ERP response showed large variability, making differentiation of the ERP components impossible. Evident and no-evident ERP components group were not significantly different in either age (*F*(1,20) = 0.002, *p* = 0.968, eta2 < 0.001) or RSSS (*F*(1,20) = 0.4545, *p* = 0.506, eta2 = 0.022). No significant differences that could be attributed to pre-processing features (e.g., number of trials (*F*(1,20) = 0.186, *p* = 0.671, eta2 = 0.009) or number of removed ICA components (*F*(1,20) = 3.274, *p* = 0.084, eta2 = 0.141)) were found between the two RS subgroups. In the current manuscript we present the data only for the Rett syndrome girls with evident ERP components that allow the assessment of the latency of the peaks. Further research is needed to dig into the problem of why such a sufficient number of RS girls do not have evident auditory ERPs, whether it relates to some uncontrolled technical issues or to dynamics/etiology of RS.

For TD and evident-ERP RS groups amplitude and latency measurements for the peaks were made using the MNE python tool and then verified by authors. The peak amplitude and latency values of the components were examined in wide time windows: P1 (33–99 ms), N1 (96–173 ms), P2 (139–264 ms), N2 (210–365 ms) consistent with previous literature and individual peaks assessments [7, 33–36]. Peak amplitudes were calculated relative to baseline and peak latencies were calculated relative to stimulus onset. Peak to peak amplitudes for N1P1, P2N1 and N2P2 were calculated as the difference in amplitude between two peaks. Considering peak to peak amplitude makes it possible to eliminate the effects caused by the contamination of the components [37–39].

The effects associated with the stimuli presentation rate and diagnosis were investigated by mixed analysis of variance (ANOVA) for the peak P1 amplitude and N1P1, P2N1 and N2P2 peak-to-peak amplitudes, and for latency of P1, N1, P2, and N2 components. Statistical analysis was performed using R-Studio software. Mixed ANOVA included Group as between-subjects factor (RS vs TD), Presentation rate (SOA—stimulus onset asynchrony) as within-subjects factor (three levels: 900 ms, 1800 and 3600 ms) and Age as covariate as well as their interactions. Estimation stats were performed using the package Dabestr [40]. Also to estimate the relations between the modulation mechanisms of the N1 and P2 components, Pearson correlation coefficient for the associated peak to peak amplitudes was calculated.

Statistical correlations between ERP components, and severity of Rett syndrome were assessed by Pearson correlation coefficients. Partial Pearson correlation coefficients, adjusting for age, were calculated for RSSS and each measurement of the ERP component that showed a significant group effect. Correlation coefficients were calculated using the ppcor library in R (www.r-package.org).

## Results

**Fig. 2:**
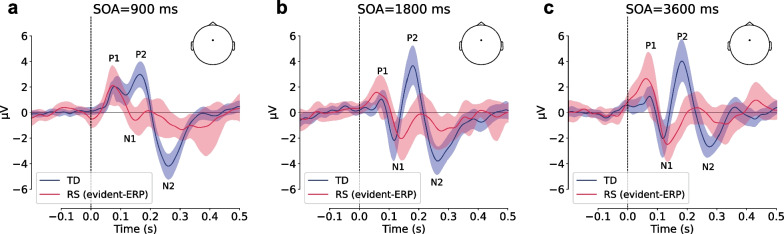
Event-related potentials (ERPs) of Typical development (TD) (blue line), and Rett syndrome (RS) (red line) groups (FCz electrode) in different stimulus onset asynchrony (SOA) conditions: **a** 900 ms, **b** 1800 ms, **c** 3600 ms. Shading corresponds to 95% confidence level

The grand-averaged ERPs in response to tones presented with different SOAs showed the expected pattern of identifiable P1, N1, P2 and N2 components in the TD participants (Fig. 2, FCz, for the topography and all channels response see Additional file 1: Fig. 1). The adaptation of the component N1 was clearly observed (its amplitude was much smaller at the shortest SOA). It is also notable that the prolongation of SOA from 1800 to 3600 ms had no impact on the evoked response component configuration. In the RS group, it was also possible to recognize the main components (especially the early ones). Comparing the evoked responses of the two groups, the early components of the ERP (P1, N1) in the RS group are relatively preserved. The later components (P2, N2), which were hardly expressed at the short SOA (900 ms), become more evident with an increase in the presentation rate but still clearly reduced and delayed than in TD. Below we prove these observations statistically. See Table 1 for the reference to the numerical values of ERP components’ amplitude and latency measures.

**Table 1:** Peak amplitudes and latencies of ERP components in TD and RS groups for different SOA conditions

Group	TD			RS		
SOA	900 ms	1800 ms	3600 ms	900 ms	1800 ms	3600 ms
*Peak amplitude, μV*						
P1	3.092 (2.146)	2.472 (2.363)	2.388 (2.45)	3.041 (2.802)	2.919 (1.995)	4.032 (2.718)
N1P1	3.876 (3.146)	5.644 (3.075)	6.464 (3.828)	4.146 (3.437)	6.17 (3.507)	8.135 (3.586)
P2N2	4.982 (4.515)	7.928 (5.61)	8.514 (5.534)	3.022 (2.83)	4.194 (3.162)	5.459 (3.45)
N2P2	8.916 (4.53)	9.335 (4.758)	8.776 (5.433)	3.983 (2.778)	3.606 (2.727)	4.017 (3.273)
*Peak latency, ms*						
P1	89.704 (18.37)	79.111 (15.653)	81.333 (15.66)	84.5 (20.593)	75.333 (27.848)	69.167 (24.987)
N1	127.481 (26.836)	122.37 (18.966)	125.259 (20.852)	139.333 (20.169)	148.5 (32.843)	139.333 (29.571)
P2	175.259 (17.958)	176.519 (16.404)	185.481 (18.327)	197.167 (28.875)	200.667 (40.203)	215.167 (32.265)
N2	253.481 (28.794)	271.185 (27.759)	276.148 (30.483)	273.333 (42.343)	276.667 (37.478)	300.167 (50.944)

*TD* typical development, *RS* Rett syndrome, *SOA* stimulus onset asynchrony

Values are shown as mean (SD)

### P1

#### P1 peak amplitude value

**Fig. 3:**
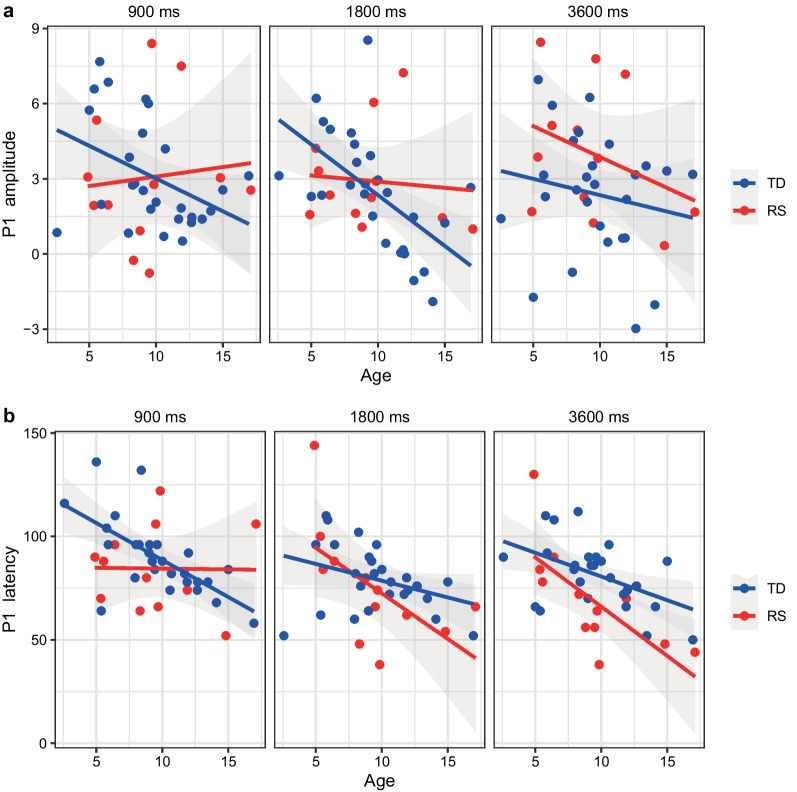
P1 characteristics: **a** P1 amplitude attenuation with age but only in typical development (TD) group at 1800-ms stimulus onset asynchrony (SOA) condition; **b** P1 latency shortening with age in all conditions in typical development (TD) but not in Rett syndrome (RS) group at 900-ms SOA condition. Dots represent individual values (blue—TD group, red—RS group)

Significant Age effect (*F*(1;35) = 4.448, *p* = 0.042, eta2 = 0.113) was found for P1 peak amplitude: P1 reduction with age. There was also significant SOA*Group interaction (*F*(2;70) = 3.264, *p* = 0.044, eta2 = 0.085) that points to the differential P1 modulation by SOA for TD and RS group (confirmed by Group effect for difference between 900 and 3600 ms SOA condition (*F*(1;38) = 4.752, *p* = 0.036, eta2 = 0.114) as P1 decrease for TD and increase in RS with prolongation of SOA (Table 1), for the full post-hoc comparison see Additional file 1: Table S3). SOA*Group*Age (*F*(2;70) = 3.758, *p* = 0.028, eta2 = 0.097) interaction was also observed for this component. Post-hoc follow-up demonstrated that Age effect was observed only in the TD group and only for 1800-ms SOA condition (*F*(1;35) = 8.714 *p* = 0.006, eta2 = 0.199) (Fig. 3a) (For more details see Additional file 1: Table S3).

#### P1 latency value

For the P1 latency general SOA effect (*F*(2;70) = 6.478, p = 0.002, eta2 = 0.162) was found: at 900-ms SOA latency was shorter, compared to other SOAs. The main effect of Age (*F*(1;35) = 23.492, *p* < 0.001, eta2 = 0.402) reflected longer P1 latency for younger children. Additionally significant SOA*Group*Age interaction (*F*(2;70) = 6.251, *p* = 0.003, eta2 = 0.152) was observed with post-hoc indicating P1 latency shortening with age for all conditions and groups except the RS group in 900-ms SOA condition (Fig. 3b).

### N1

#### N1P1 peak-to-peak amplitude value

**Fig. 4:**
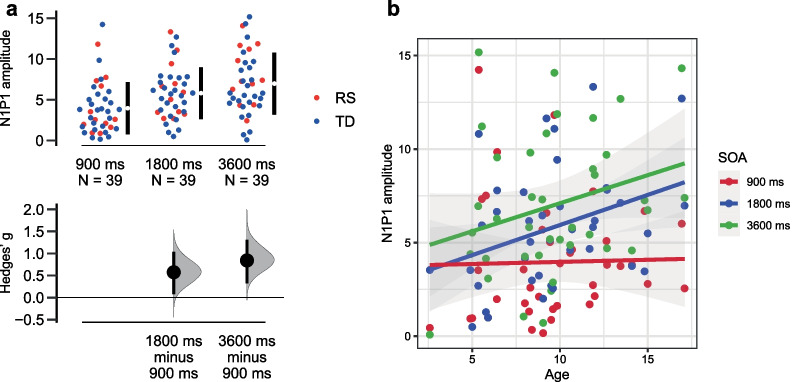
N1P1 amplitude characteristics: **a** N1P1 amplitude modulation by stimulus onset asynchrony (SOA). Dots represent individual values (blue—typical development (TD) group, red—Rett syndrome (RS) group), lower panel shows effect size (Hedges’ g); **b** SOA effect becomes more pronounced with age. Dots represent individual values in different SOA condition (red—900-ms SOA, blue—1800-ms SOA, green—3600-ms SOA)

Significant SOA effect (*F*(2;70) = 24.957, *p* < 0.001, eta2 = 0.416) was found for N1P1 amplitude. In the 900-ms SOA condition, the amplitude of this component was smaller than in the slower presentation rate. This effect was expressed in both TD and RS groups (Fig. 4a). Significant SOA*Age interaction (*F*(2;70) = 3.576, *p* = 0.033, eta2 = 0.093) represented the age-increasing difference between the 900-ms SOA and longer SOAs conditions (Fig. 4b).

#### N1 latency value

**Fig. 5:**
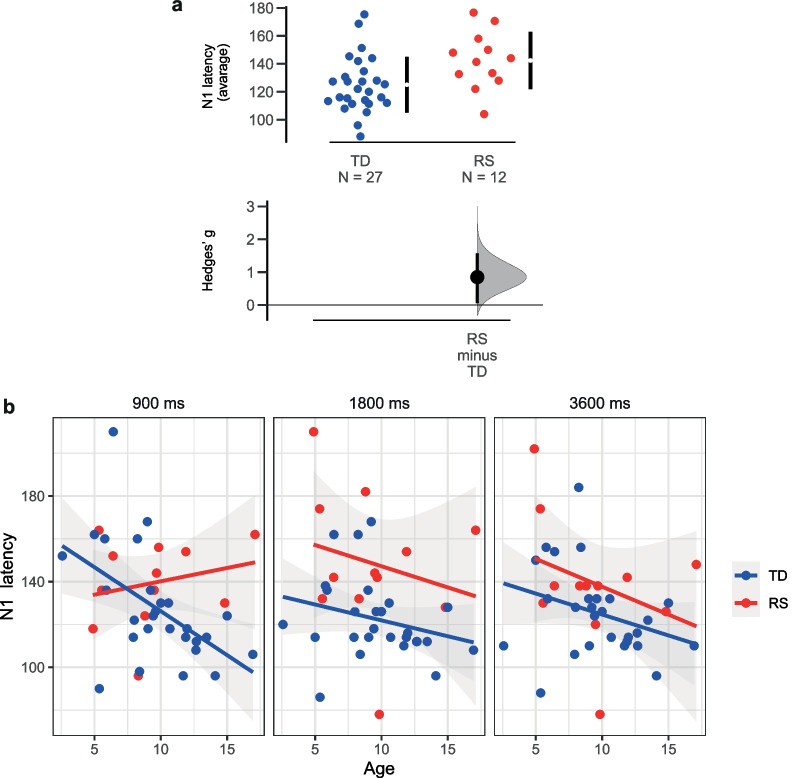
N1P1 latency characteristics: **a** Delayed N1 in Rett syndrome (RS) as compared to typical development (TD); **b** Age dynamics in TD and RS groups in different stimulus onset asynchrony conditions. Dots represent individual values (blue—TD group, red—RS group), lower panel in (**a**) shows effect size (Hedges’ g)

Significant differences between groups were found for the N1 component latency (*F*(1;35) = 6.800, *p* = 0.013, eta2 = 0.163). N1 were delayed in the RS group compared to TD (Fig. 5a). Detected significant SOA*Group*Age interaction (*F*(1;70) = 4.612, *p* = 0.013, eta2 = 0.116) demonstrated that N1 latency shortened with age, but only in the TD group and at 900-ms SOA condition (Fig. 5b).

### P2

#### P2N1 peak-to-peak amplitude value

**Fig. 6:**
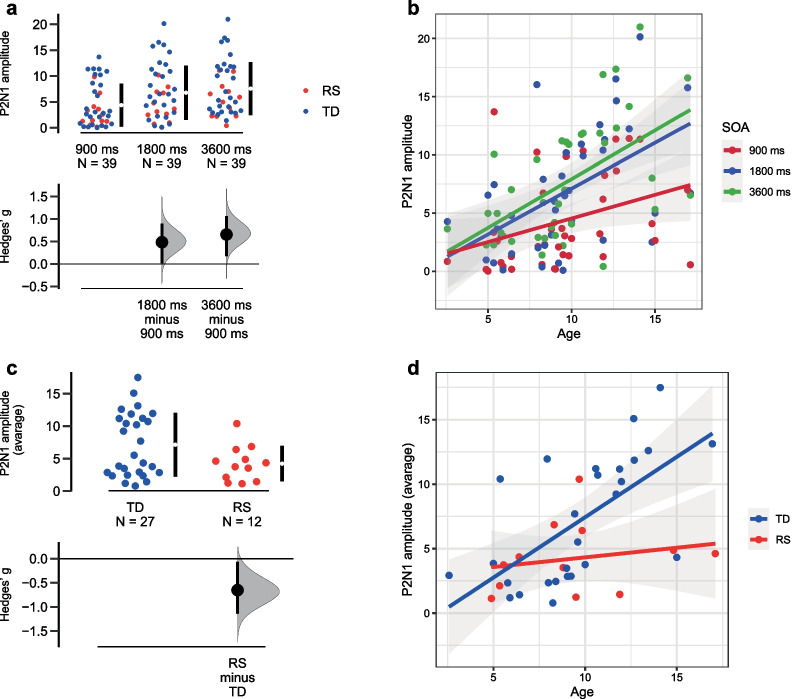
P2N1 amplitude characteristics: **a** P2N1 amplitude modulation by stimulus onset asynchrony (SOA). Dots represent individual values (blue—typical development (TD) group, red—Rett syndrome (RS) group), lower panel shows effect size (Hedges’ g); **b** SOA effect becomes more pronounced with age. Dots represent individual values in different SOA conditions (red—900-ms SOA, blue—1800-ms SOA, green—3600-ms SOA); **c** Lower P2N1 amplitude in the RS group. Dots represent individual values (blue—TD group, red—RS group), lower panel shows effect size (Hedges’ g); **d** P2N1 amplitude enlargement with age in TD, but not in RS group. Dots represent individual values (blue—TD group, red—RS group)

A significant general SOA effect was revealed (*F*(2;70) = 25.737, *p* < 0.001, eta2 = 0.301): similar to the N1P1 effect, P2N1 amplitude also become larger with SOA (Fig. 6a). Additionally P2N1 amplitude enlarges with age (*F*(1;35) = 15.093, *p* < 0.001, eta2 = 0.301). SOA*Age interaction (*F*(1;70) = 5.712, *p* = 0.005, eta2 = 0.140) demonstrates that the SOA effect becomes more pronounced with age (Fig. 6b).

Significant main effect of Group (*F*(1;35) = 5.496, *p* = 0.025, eta2 = 0.135) pointed to general amplitude reduction in the RS group as compared to TD irrespective of SOA (Fig. 6c). Additionally P2N1 amplitude enlargement with age was evident only in the TD group (Group*Age interaction (*F*(1;35) = 4.891, *p* = 0.034, eta2 = 0.123)) (Fig. 6d).

#### P2 latency value

**Fig. 7:**
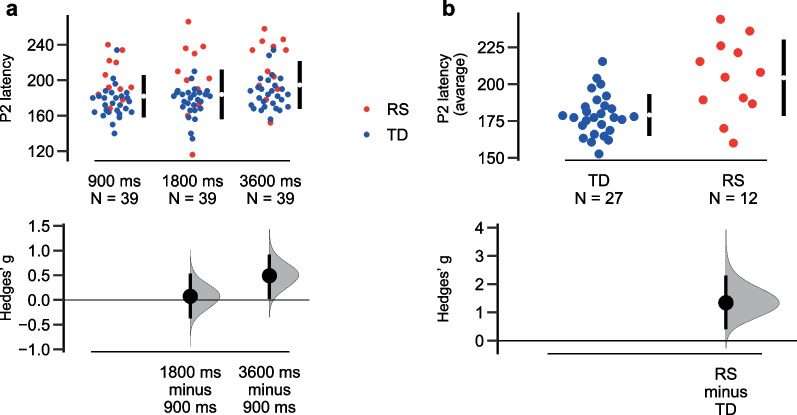
P2N1 latency characteristics: **a** P2 latency modulation by stimulus onset asynchrony (SOA); **b** Evidently delayed P2 in Rett syndrome (RS) as compared to typical development (TD). Dots represent individual values (blue—TD group, red—RS group), lower panel shows effect size (Hedges’ g)

The main effect of the SOA was detected: P2 component was significantly delayed at the slower presentation rate irrespective of group (*F*(2;70) = 5.252, *p* = 0.007, eta2 = 0.130) (Fig. 7a).

Also P2 latency was longer in the RS than in TD group (main effect of Group: *F*(1;35) = 15.272, *p* < 0.001, eta2 = 0.304) (Fig. 7b).

### N2

#### N2P2 peak-to-peak amplitude value

**Fig. 8:**
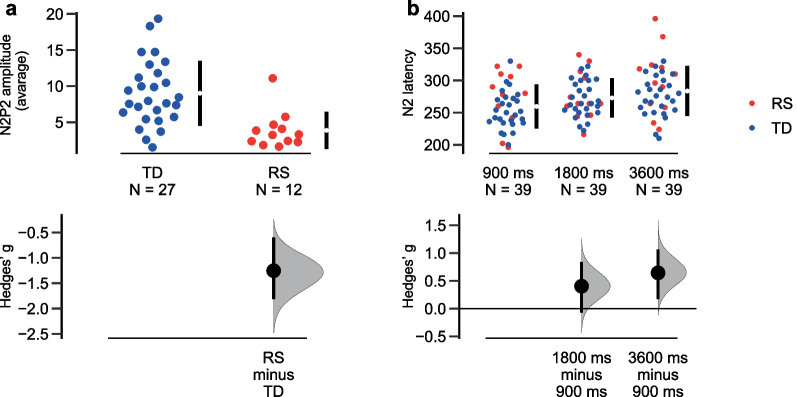
N2 characteristics: **a** Patients with Rett syndrome (RS) showed lower N2P2 amplitude; **b** The N2 latency modulation by stimulus onset asynchrony (SOA). Dots represent individual values (blue—typical development (TD) group, red—Rett syndrome (RS) group), lower panel shows effect size (Hedges’ g)

For the N2P2 component significant Group effect (*F*(1;35) = 13.506, *p* < 0.001, eta2 = 0.278) was found: N2P2 amplitude was reduced in the RS group (Fig. 8a).

#### N2 latency value

For N2 latency significant SOA effect (*F*(2;70) = 11.569, *p* < 0.001, eta2 = 0.248) pointed to the prolongation of the latency with the increase of SOA irrespective of the group (Fig. 8b).

Severity of symptomatology showed no significant correlations with neurophysiological measures that differentiated RS from TD (For more details see Additional file 1: Figs. 2 and 3).

As there is no unified view on whether the N1 and P2 components and especially their modulation by the rate of presentation represent independent processes, we examine the correlation between N1P1 and P2N1 amplitudes and their modulation by SOAs (difference of amplitudes between conditions). No significant correlations were found for the TD group, but there was a weak correlation (*R* = 0.62) between N1P1 and P2N1 amplitudes in 900-ms SOA condition in the RS group, generally supporting largely different neuronal underpinnings for these effects (For more details see Additional file 1: Figs. 4 and 5).

## Discussion

Our main goal was to examine the neurophysiological characteristics of auditory processing in girls with Rett syndrome as well as their modulation by the rate of stimulus presentation. We showed that early stages of auditory processing are relatively preserved at least in the subgroup of RS patients, while the later stages, reflected in the P2 and N2 components of ERP, are largely affected being both delayed and attenuated. At the same time, N1 and P2 components of ERP, demonstrated preserve modulation by the stimulation rate in Rett syndrome showing enlargement with the prolongation of stimulus onset asynchrony. Developmental stagnation of some neurophysiological characteristics was also observed in RS. Below we consider these findings in detail.

### Preserved modulation of ERP components by the rate of presentation in RS

In line with the previous studies [7–9] on TD, we also showed that ERP components (N1P1 and P2N1) become more pronounced with increasing stimulus onset asynchronies. The novel result is that ERPs enlargement was mostly between the SOA 900 ms and SOA 1800 ms conditions, while further prolongation in SOA up to 3600 ms did not influence ERPs. This is a rather surprising result as previous studies reported that ERP continues to increase for the SOA of at least up to 12 s, however, they were all conducted in adults [11, 12]. For the children population the studies of the effects of SOA on ERP are quite limited and none of the studies included in their design the SOA longer than 2400 ms [36, 41]. The absence of further enlargement in the ERP components after 1800 ms SOA may be related to the fact that in children the response recovery period is faster, and, as a result, sensory memory is shorter. In line with this result, it has been shown that in children, previous experience has less influence on perception [42]. However, this result needs further investigation.

An important finding is that both N1P1 and P2N1 modulation by SOA, that are largely independent, is preserved in the evident-ERP subgroup of patients with RS, even in spite of the largely altered (delayed and attenuated) P2N1 component. This result points to the typical functional meaning of these components and preservation of basic learning ability, reflected in neurophysiological adaptation in RS. As ERP components typically enlarge with the SOA prolongation, we can differentiate major ERP components in RS much better with longer SOA, suggesting that auditory information processing is less affected when tones are presented at a slow rate (e.g. with SOA 1800 and 3600 ms). However, clear between group differences is evident even with these longer SOAs.

The effect of presentation rate on auditory processing in Rett syndrome has been demonstrated previously in the oddball paradigm by means of mismatch negativity (MMN)—neurophysiological correlate of change detection assessed from ERP difference wave (standard, frequent ERP minus rare deviant ERP). MMN in response to frequency deviation has been registered in girls with RS only with a short (450 ms) interval between stimuli, but not with a longer stimulus onset asynchrony (900 ms and 1800 ms) [29]. This might indicate that the stimulus representation in Rett syndrome persists for a shorter period of time and vanishes with increasing SOA. In the present study, we have found that as SOA increases from 900 to 1800 ms, N1P2 and P2N1 amplitudes in RS become larger, indicating typical release from adaptation, which at first glance is inconsistent with these MMN data. However, the neuronal mechanisms that underlie MMN and N1/P2 components most likely are different with the MMN linked to predictive error processing and the N1/P2 associated with stimulus-specific adaptation (SSA) [43–45]. Thus, the results obtained in the present and Brima's work may indicate changes in different subprocesses of auditory processing and features of different neuronal populations’ activation in RS.

Thus, choice of the most appropriate SOA for future auditory ERP studies should be driven mainly by the process of interest, with longer SOA more appropriate for tracing change detection deficits and shorter SOA being more sensitive for alternation of basic auditory components. While speculative, such neurophysiological abnormalities together with clearly prolonged timing of information processing result in a very limited time window within which the RS brain processes information more or less adequately. Clinical field might potentially benefit from further work in collaboration with practitioners aiming to develop the training/information presentation protocols for RS that allow: (1) information that needs to be linked together being demonstrated within a short time frame and at the same time (2) provides enough time for the information processing that is clearly prolonged in RS.

We believe that other SOA effects, revealed in our study, do not represent the meaningful insight into the core deficits in RS, as they can be explained by contamination of ERP components, as enlargement in one component can lead to latency shift and reduction in the neighboring components of opposite polarity. In particular, a heightening of the N1 amplitude with SOA prolongation could lead to (1) delay in the next components (P2), which appeared as an increase in its latency with increasing SOA as well as (2) shortening of the previous component latency, which appears as P1 latency decrease with SOA prolongation. Similarly, an enlargement in the amplitude of P2 leads to a prolongation in the latency of the following N2 component.

### Delayed and reduced ERP components in RS

In our study significant reduction in the amplitude was shown for P2 (P2N1) and N2 (N2P2) components in RS. Also, the P2 and N1 components were significantly delayed in the RS group compared to TD.

Previous studies describing auditory evoked potentials in patients with Rett syndrome often highlight their attenuation and delay in comparison to typically developing children [16, 18, 29]. In particular, Sysoeva's study demonstrated a reduction in the amplitude of the P2 and N2 components of auditory ERP in response to simple (tones) and more complex (phonemes) types of stimuli [15], with the former being confirmed in our current study. An atypical reduction of P2 measured as P2N1 amplitude was also reported in a multisite study of Saby and colleagues, however, unlike our results their RS patients showed also a reduction in N1 amplitude (N1P1 peak-to-peak) [16]. This discrepancy may be due to the slightly different experimental conditions (e.g. varied SOA from 0.6 to 2.0 s) or the wider age range of their patients (2–37 years old), since SOA variability as well as age was shown to increase N1 amplitude [35].

At the cognitive level these effects can be linked to a disturbance of information processing in the late stages. The P2 component is associated with the consolidation in the auditory memory [21]. Meanwhile, N2 is associated with the inhibition of irrelevant information [26] and categorization [22–25]. Thus, a reduction in both of these components in Rett syndrome may be related to the alternation in these abilities.

However, the deficits start at the level of the N1 component, which is significantly delayed in Rett syndrome. Such latency shift suggests the increased processing time needed for auditory stimuli identification. For the first time, changes in the auditory ERPs’ latencies in Rett syndrome have been described by Bader in 1989 who reported some patients with RS demonstrating the delay of Pa, N1 and P2 components at the individual level [46]. Delay in P2 component has been also confirmed in subsequent studies [15], including our current work. The MMN, that coincides in latency with the N1 component (circa 120 ms), has been also shown to be delayed in Rett syndrome as compared to the control group [18]. While ERP’s latencies shift in RS in our and previous studies is only about 20–40 ms, information processing in the auditory system is extremely fast, making even small temporal delays critical, especially for complex stimuli processing such as speech.

Noteworthy, the ERP abnormalities similar to what we found in patients with RS is also observed in RS animal models: *Mecp2*-deficient mice and rats demonstrated delayed and reduced analogous auditory cortex response [47–49]. Moreover, ERP component delay is evident in RS not only in auditory modality, but also observed in response to visual stimuli [50–52]. So this pattern is quite consistent for Rett syndrome and related *Mecp2* damages.

### Developmental course of ERP components and their relationship with Rett syndrome severity

We have found atypical age dynamics (stagnation) for several neurophysiological characteristics in patients with RS. TD group in our study characterized by developmental decrease in the amplitude and latency of P1, an increase in the amplitude of N1P1 and a shortening of the latency of N1, as well as an increase in the amplitude of P2N1, which corresponds to the previous literature [7, 33–36]. Some age-effects were more pronounced for particular SOA conditions, generally corresponding to longer SOAs, where components are larger. Modulation by presentation rate of N1P1 and P2N1 amplitudes also show increase with age, corresponding with previous results [35]. Among these developmental changes only N1P1 amplitude followed typical developmental increase in RS. All other components showed atypical age dynamics, e.g. full developmental stagnation irrespective to SOAs for P2N1, or absence of age-related decrease in P1 latency specific only to 900-ms SOA condition with preserved P1 latency decreased for longer SOAs. Thus, this atypical age dynamics of neurophysiological components corresponds well with the view on the Rett syndrome as a disorder of stagnated development also seen at the behavioral level.

The absence of a significant association with age in Rett syndrome, in contrast to the TD group, has been described previously by Sysoeva for 900-ms SOA condition. This work demonstrated the age-dependent increase in N1 and decrease in P1 and N2 components in the TD group, but not in the RS group, but this result was not adequately powered to detect between-group differences in the developmental trajectory [15]. Differences in age dynamics between the groups have been described by Saby et al., but in this work Rett syndrome group demonstrated a decline in N1P1 peak to peak amplitude with age which was not observed in the TD group [16]. It was suggested that this negative dynamic could represent a progressive aspect of the disease process. Despite the fact that we found the typical increase of these components (P1, N1) peak-to-peak values in RS group in our sample, which could be caused by differences in the experimental procedure and a narrow age range, our results also indicate altered auditory ERP development, especially for P2N1, which was reduced in the RS group.

Multiple papers [15, 16, 52] demonstrated correlation of ERP amplitudes or latency to Rett syndrome severity. In Saby’s work Clinical Severity Score (CSS [53, 54]) and Motor-Behavioral Assessment (MBA [54]) were used [16]. Both of these scales showed significant correlation with N1 amplitude and N1P1 peak-to-peak amplitude. Sysoeva et al. used the same RSSS as in our study and observed significant correlation with P2 amplitude [15]. In our study, no significant correlations between ERP components and RSSS were found. The absence of significant correlations may be due to the coarseness of the chosen scale or to some difficulties in adapting it for the Russian populations.

### Approaches to no-evident ERP group

It is a continuous struggle for researchers, especially those who work with a challenging population, to include as much data as possible preferentially without compromising on signal to noise ratio (SNR). However, quite often a lot of data needed to be excluded from the final sample due to various, sometimes subjective, reasons. For example, if you exclude participants without evident ERP components—that seems reasonable as it might represent some problems with EEG recording—you might exclude the patients that indeed have very small and not very pronounced ERP and this absence of evident ERP might be important characteristics of the population considered. Our sample contained about half of the RS group without evident ERPs, and we selected for the main analysis only those who have evident ERPs with measurable ERP components. Abnormalities that are revealed in the evident-ERP group cannot be attributed to the poor SNR or other technical problems, and considered in our study as the genuine neurophysiological atypicalities that characterize RS. Moreover, in this group we can examine not only the amplitude but also latency of the ERP components providing a more in depth view on the origin of the observed changes.

The causes of the fact that only half of the participants with Rett syndrome had evident components are currently unclear, but the phenomenon itself corresponds with previous studies in RS [16, 18, 29, 52]. This specific pattern was not due to age and the severity of the symptoms of the disease. One of the possible causes of this could be abnormal background EEG, which has always been considered one of the features of this syndrome [55, 56]. The epileptiform activity in Rett syndrome is expressed by the appearance of spikes and sharp waves in the central temporal regions and a slowing of the theta-band background EEG in the frontal-central regions [17, 57–59]. These features are unrelated to seizures and occur even in patients without a history of epilepsy [57, 58, 60, 61]. Thus, against the background of an epileptiform activity or just increased low-frequency oscillations, the detection of evoked potentials could be problematic. Whether this “ERP absence” is a true characteristic of some RS patients or a result of technical problems in detection of ERPs in the presence of atypical background EEG activity is an important direction for future research.

## Limitations

Several limitations should be mentioned when interpreting our findings. As we could not figure out the cause for such a high percentage of no-evident ERP RS participants, this issue has to be further investigated. For example, it could be a result of some uncontrolled parameters, such as time of experiment and tiredness of the participant. Thus, the final RS group was of rather small size that limited the generalizability of the results. Also, we used a high-pass filter that could potentially alter the results. Examination of RS behavioral phenotype can be also improved by usage/developing more detailed tools. It is important to point out the absence of a control clinical group. This contrast would allow us to distinguish whether the findings are actually neuromarkers specifically for Rett syndrome or shared with other neurological or psychiatric conditions.

## Conclusions

To sum up, the stimulus onset asynchrony modulates the amplitude and latency of the main auditory ERP components similarly in typical development and in Rett syndrome, suggesting preserved basic neuronal learning mechanisms (adaptation) at least at the subgroup of patients with RS with evident auditory ERP. At the same time, even in this group the characteristics of auditory ERP are clearly disturbed, such as N1 and P2 components are delayed and P2 and N2 amplitudes are attenuated and abnormalities persist even at the slowest presentation rate. Such neurophysiological changes suggest deficits at the speed and quality of auditory processing in RS. Moreover, most characteristics of auditory ERP do not show typical developmental changes with age in RS, corresponding to the view on RS as a disorder of stagnated development.

### Supplementary Information

**Additional file 1 **contains supplementary tables and a supplementary figure.

## Data Availability

The datasets used and analysed during the current study are available from the corresponding author on reasonable request.

## Abbreviations

ANOVA: Analysis of variance
EEG: Electroencephalography
ERP: Auditory event related potentials
Hz: Hertz
ICA: Independent component analysis
MMN: Mismatch negativity
RS: Rett syndrome
RSSS: Rett syndrome severity scale
SNR: Signal to noise ratio
SOA: Stimulus onset asynchrony
SSA: Stimulus specific adaptation
TD: Typical development
