# Supplementary material for: Effect of presentation rate on auditory processing in Rett syndrome: event-related potential study
Source: Mol Autism. 2023 Oct 26;14:40. doi: 10.1186/s13229-023-00566-1 (PMC10605980; doi:10.1186/s13229-023-00566-1)

**SUPPLEMENTARY MATERIAL**

**Supplementary Table 1.** Characteristic of the RS sample

| ID | Regress Age | RSSS:Breath | RSSS: Speech | Total RSSS | ERP | Age | Type of mutation |
| --- | --- | --- | --- | --- | --- | --- | --- |
| R001 | 18 | 2 | 3 | 12 | evident | 5,55 | МEСР2 c.763 C>T, р.R255X |
| R002 | 24 | 0 | 2 | 5 | evident | 6,40 | МEСР2 c.953G>A, p.R318H |
| R006 | 15 | 1 | 2 | 6 | no-evident | 7,50 | МEСР2 с.502С>Т, p.R168X |
| R015 | 12 | 1 | 2 | 9 | evident | 5,34 | МEСР2 с.730С>T, p.Q244 |
| R016 | 26 | 0 | 2 | 4 | evident | 8,80 | МEСР2 c.397C>T, p.R133C |
| R017 | 15 | 3 | 3 | 4 | evident | 4,89 | МEСР2 с.352С>T, p.R118W |
| R018 | 18 | 2 | 3 | 14 | evident | 14,82 | МEСР2 с.115del144р |
| R020 | 27 | 3 | 3 | 14 | evident | 17,10 | del Xq28 (including МEСР2) |
| R022 | 17 | 0 | 3 | 12 | no-evident | 9,88 | МEСР2 c.808C>, p.R270X |
| R023 | 8 | 0 | 2 | 10 | no-evident | 6,27 | МЕCP2 c.763C>T, p.R255X |
| R024 | 21 | 3 | 3 | 13 | no-evident | 6,65 | МEСР2.952C>T, p.R318C |
| R026 | 30 | 1 | 3 | 18 | no-evident | 12,04 | МEСР2 с.889C>T, p.R297X |
| R027 | 30 | 3 | 3 | 8 | evident | 8,31 | МEСР2 с.1164_1207del44, p.P388fs |
| R028 | 15 | 1 | 2 | 11 | no-evident | 10,17 | МEСР2 c.808C>T, p.R270X |
| R029 | 10 | 1 | 3 | 11 | no-evident | 15,62 | МEСР2 c.880С>T, p.R294X |
| R030 | 15 | 0 | 3 | 11 | evident | 11,89 | МEСР2 c.423С>G, p.Tyr141X |
| R031 | 15 | 0 | 2 | 5 | no-evident | 15,96 | МEСР2 с.753delC, p.G252fs |
| R032 | 18 | 1 | 2 | 12 | no-evident | 6,00 | МEСР2 c.844C>T, p.R282X |
| R034 | 7 | 0 | 3 | 12 | evident | 9,68 | МEСР2 с.502С>Т/N, р.R168X |
| R036 | 11 | 2 | 3 | 13 | evident | 9,84 | МEСР2 с.63-6C>G |
| R037 | 16 | 3 | 2 | 13 | evident | 9,50 | МEСР2 c.763С>T, p.R255X |
| R041 | 11 | 3 | 3 | 12 | no-evident | 4,03 | МEСР2 c.844C>T, p.R282X |

Rett Syndrome Severity Scale (RSSS) assesses individual parameters: frequency and severity of seizures, breathing irregularities, scoliosis, ability to walk, use of hands, speech and sleep. For RSSS each parameter is rated on a Likert scale ranging from 0 (none/normal) to 3 (severe), with a maximum value of 21 points. The table presents the scores for the most relevant RSSS subscales and the total RSSS scores. The ERP column presents the results of the assessment of the evidence of ERP components by experts.

**Supplementary Fig. 1** Butterfly plot and peaks topomaps of TD and RS group ERPs in (a,b) 900 ms, (c, d) 1800 ms and (e, f) 3600 ms SOA condition


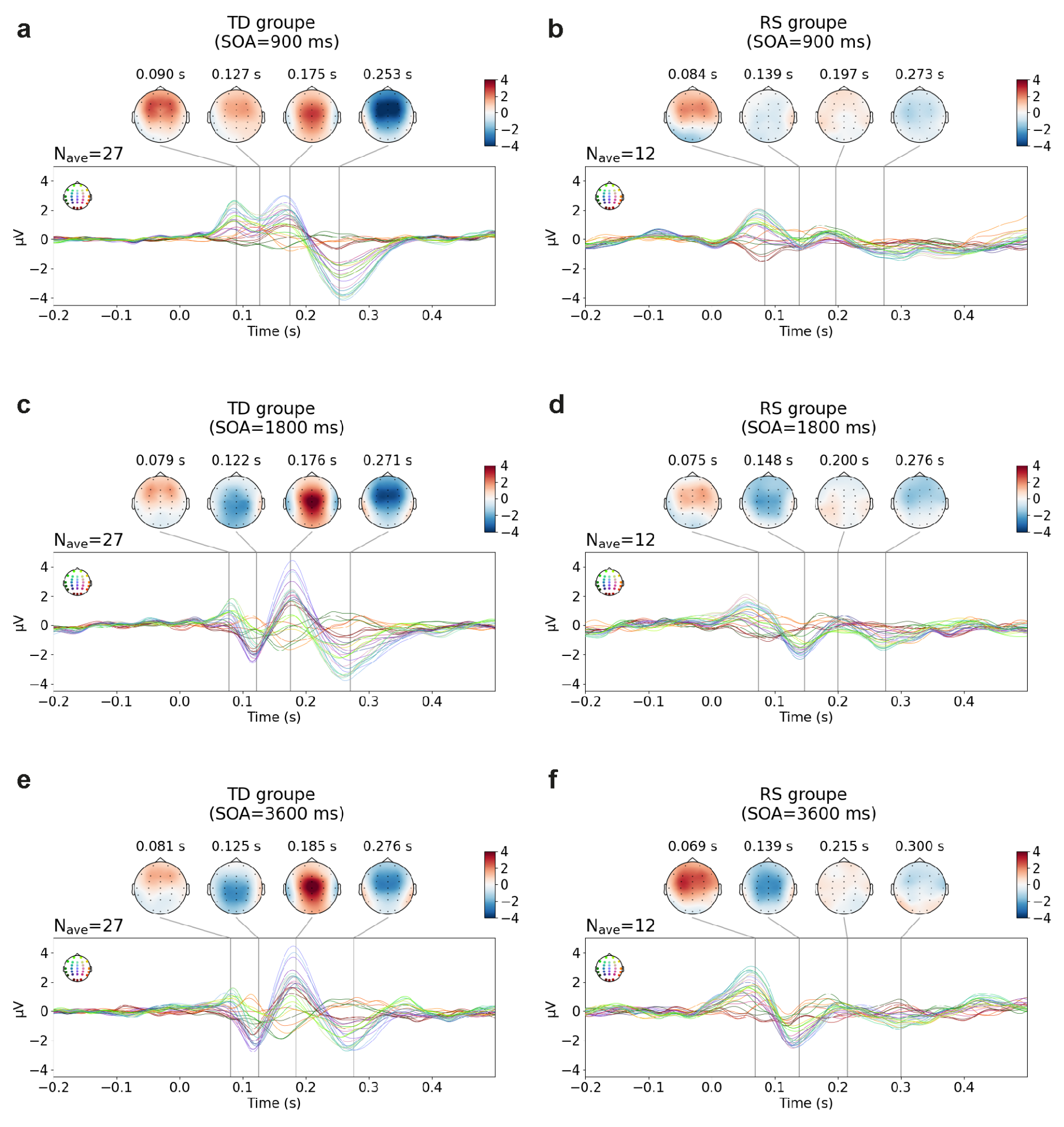


**Supplementary Table 2.** Main ANOVA results

|  | Effect | DFn | DFd | F | p | partial eta^2 |
| --- | --- | --- | --- | --- | --- | --- |
| P1 latency | Group | 1 | 35 | 2.966 | 9.40e-02 | 0.078 |
|  | **Age** | **1** | **35** | **23.492** | **2.55e-05** | **0.402** |
|  | **SOA** | **2** | **70** | **6.784** | **2.00e-03** | **0.162** |
|  | Group:Age | 1 | 35 | 0.249 | 6.21e-01 | 0.007 |
|  | SOA:Group | 2 | 70 | 0.817 | 4.46e-01 | 0.023 |
|  | SOA:Age | 2 | 70 | 0.377 | 6.87e-01 | 0.011 |
|  | **SOA:Group:Age** | **2** | **70** | **6.251** | **3.00e-03** | **0.152** |
| N1 latency | **Group** | **1** | **35** | **6.800** | **0.013** | **0.163** |
|  | **Age** | **1** | **35** | **4.979** | **0.032** | **0.125** |
|  | SOA | 2 | 70 | 0.089 | 0.915 | 0.003 |
|  | Group:Age | 1 | 35 | 0.547 | 0.465 | 0.015 |
|  | SOA:Group | 2 | 70 | 1.892 | 0.158 | 0.051 |
|  | SOA:Age | 2 | 70 | 0.174 | 0.841 | 0.005 |
|  | **SOA:Group:Age** | **2** | **70** | **4.612** | **0.013** | **0.116** |
| P2 latency | **Group** | **1** | **35** | **15.272** | **0.000407** | **0.304000** |
|  | Age | 1 | 35 | 0.006 | 0.937000 | 0.000184 |
|  | **SOA** | **2** | **70** | **5.252** | **0.007000** | **0.130000** |
|  | Group:Age | 1 | 35 | 1.328 | 0.257000 | 0.037000 |
|  | SOA:Group | 2 | 70 | 0.389 | 0.679000 | 0.011000 |
|  | SOA:Age | 2 | 70 | 1.049 | 0.356000 | 0.029000 |
|  | SOA:Group:Age | 2 | 70 | 0.040 | 0.961000 | 0.001000 |
| N2 latency | Group | 1 | 35 | 2.871 | 9.90e-02 | 0.076 |
|  | Age | 1 | 35 | 1.916 | 1.75e-01 | 0.052 |
|  | **SOA** | **2** | **70** | **11.569** | **4.56e-05** | **0.248** |
|  | Group:Age | 1 | 35 | 3.220 | 8.10e-02 | 0.084 |
|  | SOA:Group | 2 | 70 | 1.619 | 2.05e-01 | 0.044 |
|  | SOA:Age | 2 | 70 | 0.825 | 4.42e-01 | 0.023 |
|  | SOA:Group:Age | 2 | 70 | 0.419 | 6.59e-01 | 0.012 |
| P1 amplitude | Group | 1 | 35 | 0.974 | 0.330 | 0.027 |
|  | **Age** | **1** | **35** | **4.448** | **0.042** | **0.113** |
|  | SOA | 2 | 70 | 1.116 | 0.333 | 0.031 |
|  | Group:Age | 1 | 35 | 0.956 | 0.335 | 0.027 |
|  | **SOA:Group** | **2** | **70** | **3.264** | **0.044** | **0.085** |
|  | SOA:Age | 2 | 70 | 1.216 | 0.302 | 0.034 |
|  | **SOA:Group:Age** | **2** | **70** | **3.758** | **0.028** | **0.097** |
| N1P1 amplitude | Group | 1 | 35 | 0.634 | 4.31e-01 | 0.018 |
|  | Age | 1 | 35 | 2.454 | 1.26e-01 | 0.066 |
|  | **SOA** | **2** | **70** | **24.957** | **6.58e-09** | **0.416** |
|  | Group:Age | 1 | 35 | 0.056 | 8.15e-01 | 0.002 |
|  | SOA:Group | 2 | 70 | 1.276 | 2.86e-01 | 0.035 |
|  | **SOA:Age** | **2** | **70** | **3.576** | **3.30e-02** | **0.093** |
|  | SOA:Group:Age | 2 | 70 | 1.961 | 1.48e-01 | 0.053 |
| P2N1 amplitude | **Group** | **1** | **35** | **5.476** | **2.50e-02** | **0.135** |
|  | **Age** | **1** | **35** | **15.093** | **4.35e-04** | **0.301** |
|  | **SOA** | **2** | **70** | **25.737** | **4.18e-09** | **0.424** |
|  | **Group:Age** | **1** | **35** | **4.891** | **3.40e-02** | **0.123** |
|  | SOA:Group | 2 | 70 | 1.588 | 2.12e-01 | 0.043 |
|  | **SOA:Age** | **2** | **70** | **5.712** | **5.00e-03** | **0.140** |
|  | SOA:Group:Age | 2 | 70 | 0.451 | 6.39e-01 | 0.013 |
| N2P2 amplitude | **Group** | **1** | **35** | **13.506** | **0.00079** | **0.278** |
|  | Age | 1 | 35 | 1.319 | 0.25900 | 0.036 |
|  | SOA | 2 | 70 | 0.137 | 0.87200 | 0.004 |
|  | Group:Age | 1 | 35 | 0.399 | 0.53200 | 0.011 |
|  | SOA:Group | 2 | 70 | 0.443 | 0.64400 | 0.012 |
|  | SOA:Age | 2 | 70 | 2.048 | 0.13700 | 0.055 |
|  | SOA:Group:Age | 2 | 70 | 1.236 | 0.29700 | 0.034 |

**Supplementary Table 3.** Post-hoc results for SOA:Group:Age interaction effects.

|  |  | Effect | DFn | DFd | F | p | partial eta^2 |
| --- | --- | --- | --- | --- | --- | --- | --- |
| P1 amplitude by Group | RS | Age | 1 | 10 | 0.153 | 0.704 | 0.015 |
|  |  | SOA | 2 | 20 | 2.571 | 0.101 | 0.205 |
|  |  | SOA:Age | 2 | 20 | 2.375 | 0.119 | 0.192 |
|  | TD | **Age** | **1** | **25** | **6.184** | **0.020** | **0.198** |
|  |  | SOA | 2 | 50 | 1.986 | 0.148 | 0.074 |
|  |  | SOA:Age | 2 | 50 | 2.727 | 0.075 | 0.098 |
| P1 amplitude by SOA | 900 ms | Group | 1 | 35 | 0.004 | 0.949 | 0.000119 |
|  |  | Age | 1 | 35 | 1.724 | 0.198 | 0.047000 |
|  |  | Group:Age | 1 | 35 | 2.183 | 0.148 | 0.059000 |
|  | 1800 ms | Group | 1 | 35 | 0.413 | 0.525 | 0.012 |
|  |  | **Age** | **1** | **35** | **8.714** | **0.006** | **0.199** |
|  |  | Group:Age | 1 | 35 | 3.204 | 0.082 | 0.084 |
|  | 3600 ms | Group | 1 | 35 | 3.525 | 0.069 | 0.091 |
|  |  | Age | 1 | 35 | 2.053 | 0.161 | 0.055 |
|  |  | Group:Age | 1 | 35 | 0.214 | 0.646 | 0.006 |
| P1 latency by Group | RS | **Age** | **1** | **10** | **7.474** | **0.021** | **0.428** |
|  |  | SOA | 2 | 20 | 1.917 | 0.173 | 0.161 |
|  |  | SOA:Age | 2 | 20 | 2.871 | 0.080 | 0.2 |
|  | TD | **Age** | **1** | **25** | **15.758** | **0.000536** | **0.387** |
|  |  | **SOA** | **2** | **50** | **6.117** | **0.004000** | **0.197** |
|  |  | SOA:Age | 2 | 50 | 2.091 | 0.134000 | 0.077 |
| P1 latency by SOA | 900 ms | Group | 1 | 35 | 0.810 | 0.374 | 0.023 |
|  |  | **Age** | **1** | **35** | **8.903** | **0.005** | **0.203** |
|  |  | **Group:Age** | **1** | **35** | **4.510** | **0.041** | **0.114** |
|  | 1800 ms | Group | 1 | 35 | 0.374 | 0.545 | 0.011 |
|  |  | **Age** | **1** | **35** | **9.540** | **0.004** | **0.214** |
|  |  | Group:Age | 1 | 35 | 2.494 | 0.123 | 0.067 |
|  | 3600 ms | Group | 1 | 35 | 5.282 | 0.028000 | 0.131 |
|  |  | **Age** | **1** | **35** | **19.205** | **0.000102** | **0.354** |
|  |  | Group:Age | 1 | 35 | 2.675 | 0.111000 | 0.071 |
| N1 latency by Group | RS | Age | 1 | 10 | 0.443 | 0.521 | 0.042 |
|  |  | SOA | 2 | 20 | 0.619 | 0.549 | 0.058 |
|  |  | SOA:Age | 2 | 20 | 1.236 | 0.312 | 0.110 |
|  | TD | **Age** | **1** | **25** | **5.416** | **0.028** | **0.178** |
|  |  | SOA | 2 | 50 | 1.219 | 0.304 | 0.046 |
|  |  | **SOA:Age** | **2** | **50** | **3.907** | **0.027** | **0.135** |
| N1 latency by SOA | 900 ms | Group | 1 | 35 | 2.262 | 0.142 | 0.061 |
|  |  | **Age** | **1** | **35** | **4.304** | **0.045** | **0.110** |
|  |  | **Group:Age** | **1** | **35** | **5.672** | **0.023** | **0.139** |
|  | 1800 ms | **Group** | **1** | **35** | **9.945** | **0.003** | **0.221** |
|  |  | Age | 1 | 35 | 2.155 | 0.151 | 0.058 |
|  |  | Group:Age | 1 | 35 | 0.044 | 0.835 | 0.001 |
|  | 3600 ms | Group | 1 | 35 | 3.072 | 0.088 | 0.081 |
|  |  | Age | 1 | 35 | 3.986 | 0.054 | 0.102 |
|  |  | Group:Age | 1 | 35 | 0.079 | 0.780 | 0.002 |

**Supplementary Fig. 2** Correlation between RSSS and (a) P2N1 average amplitude; (b) N2P2 average amplitude


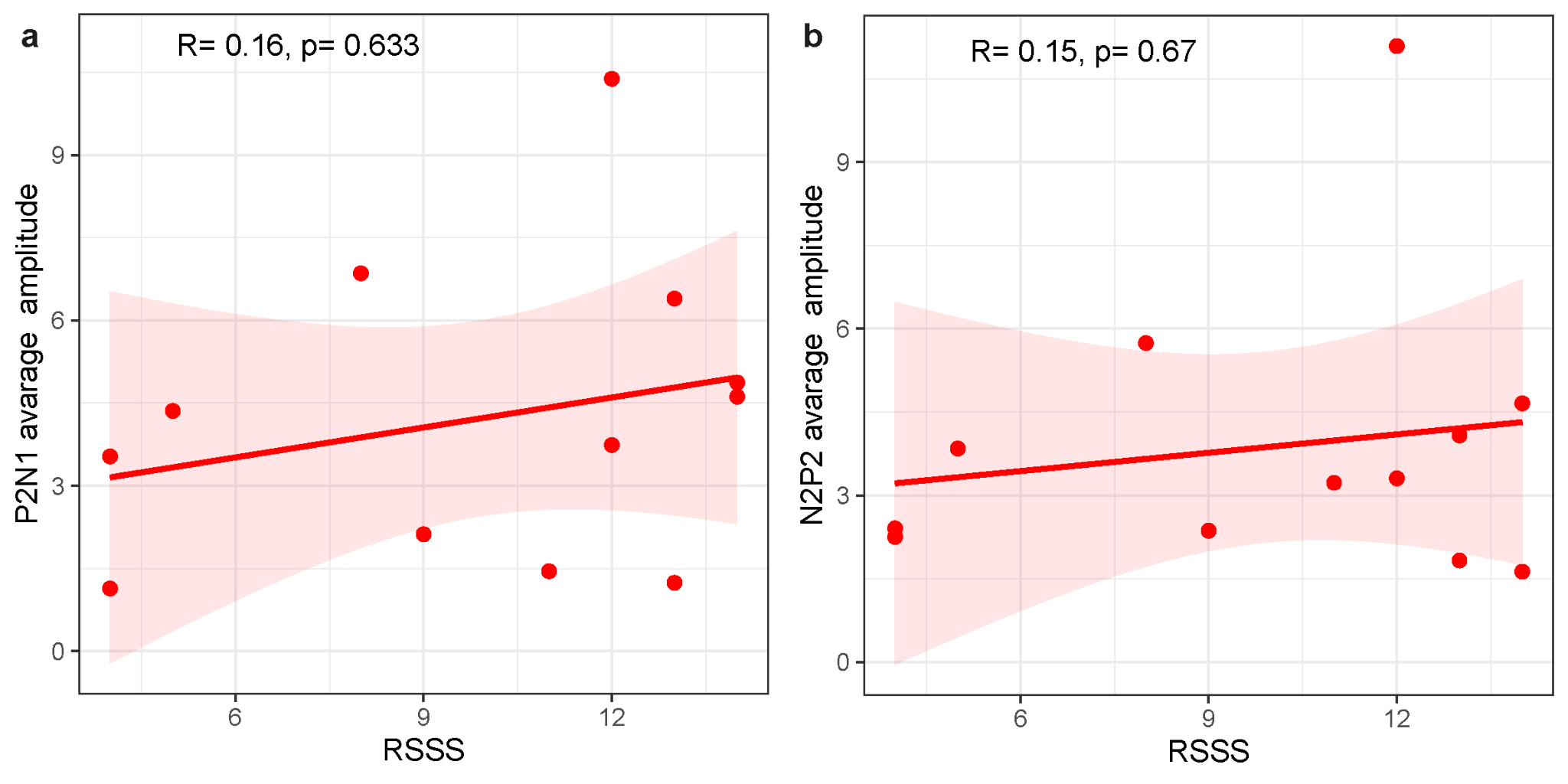


**Supplementary Fig. 3** Correlation between RSSS and (a) N1 average latency; (b) P2 average latency


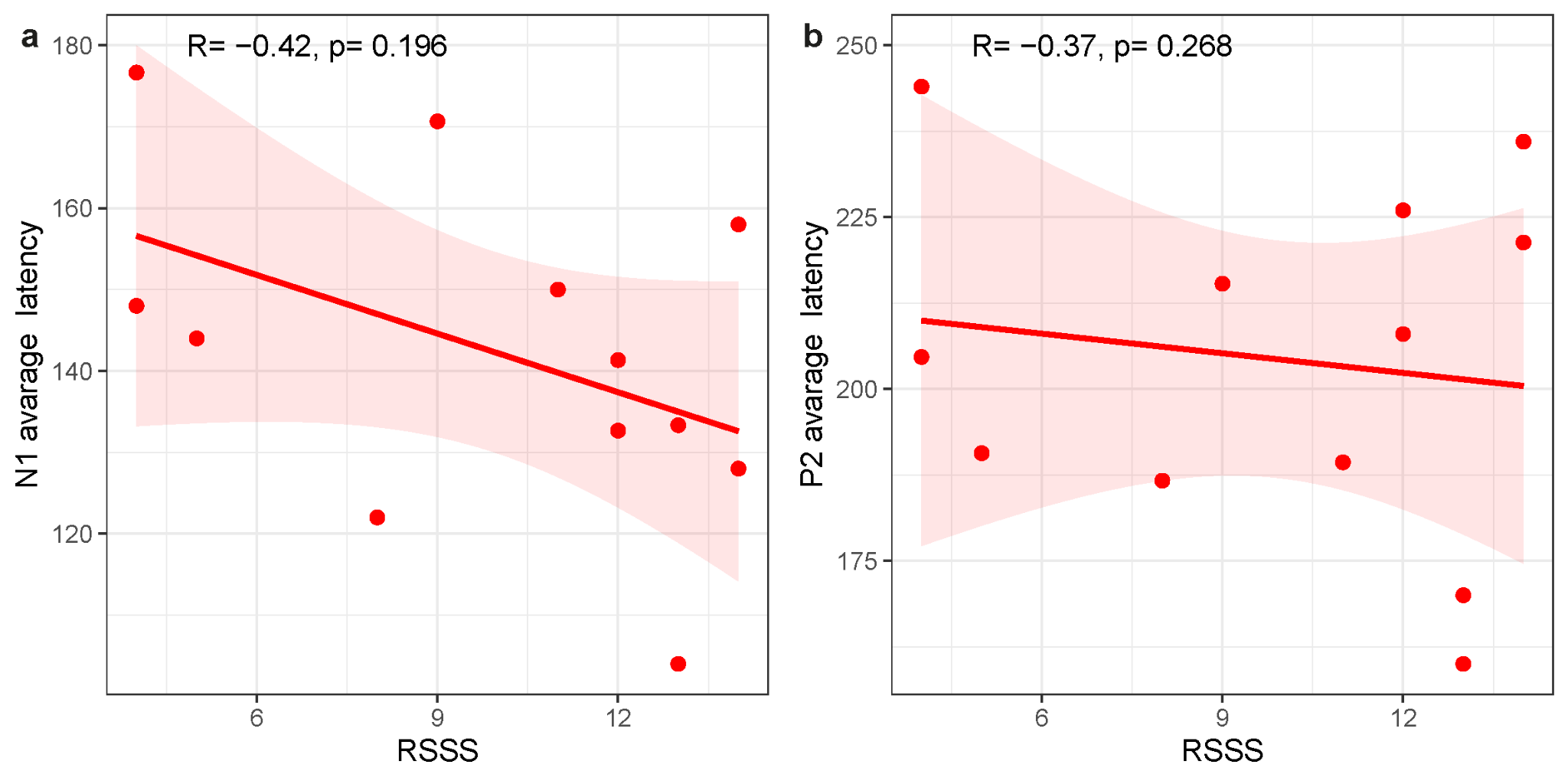


**Supplementary Fig. 4** Correlation between N1P1 and P2N1 amplitude in TD and RS groups in different SOA conditions. Dots represent individual values in different SOA conditions (red - 900-ms SOA, blue - 1800-ms SOA, green - 3600-ms SOA)


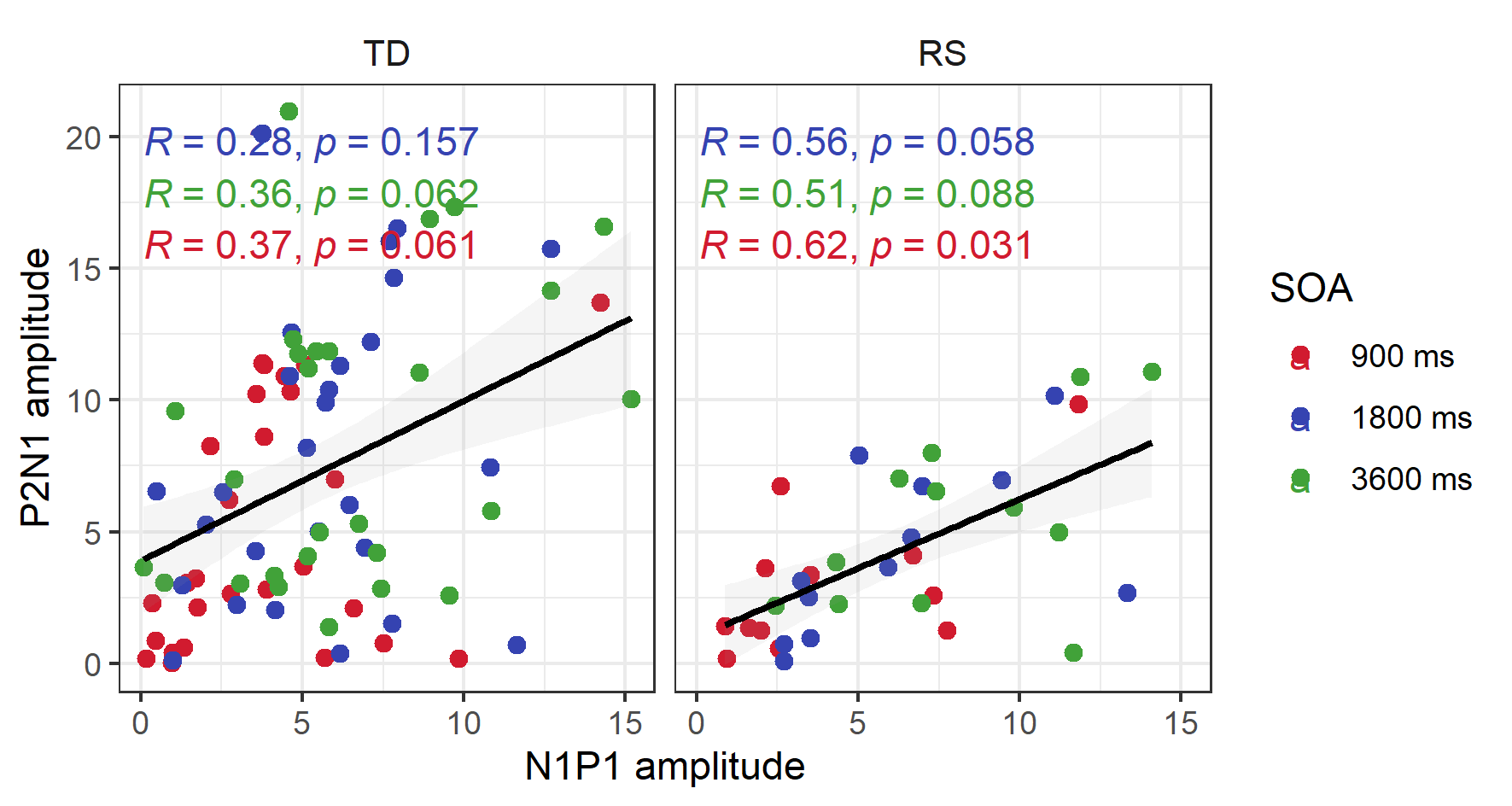


**Supplementary Fig. 5** Correlation between N1P1 and P2N1 amplitude (the difference value between 900 and 3600 conditions) in TD and RS groups. Dots represent individual values (blue - TD group, red - RS group).


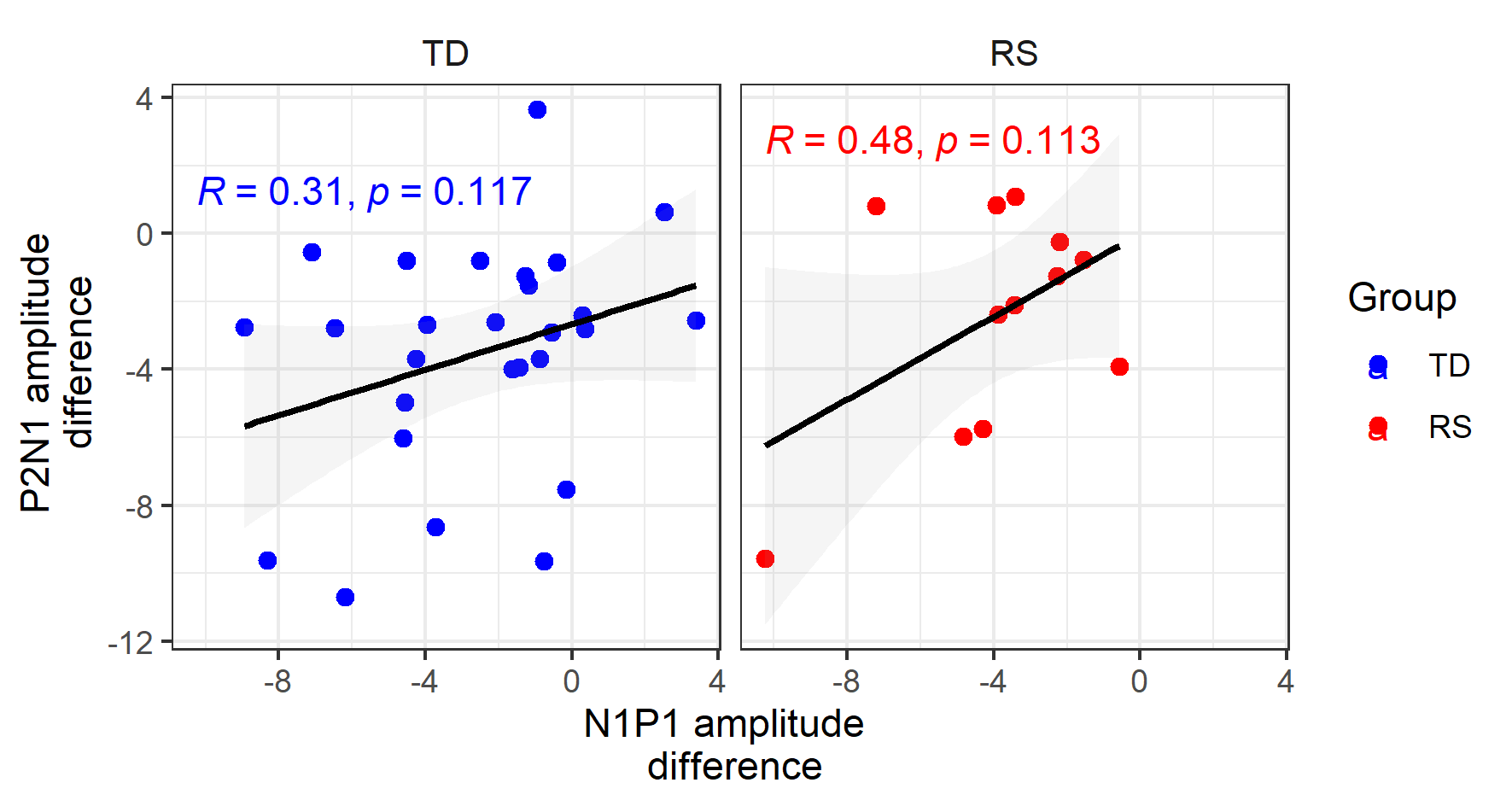

Supplement: Supplementary file 1 — Additional file 1 contains supplementary tables and a supplementary figure. [file 13229_2023_566_MOESM1_ESM.docx]
